# Disseminated Melioidosis Complicated by Dural Venous Sinus Thrombosis: A Case Report

**DOI:** 10.7759/cureus.114511

**Published:** 2026-08-13

**Authors:** Sugenthiran Muagan, Wei Xuan Tuang, Chee Yik Chang

**Affiliations:** 1 Infectious Diseases, Hospital Sultanah Aminah, Johor Bahru, MYS

**Keywords:** burkholderia pseudomallei, dural venous sinus thrombosis, melioidosis, septic emboli, splenic abscess

## Abstract

Melioidosis is a potentially fatal endemic infection caused by the Gram-negative bacterium *Burkholderia pseudomallei*. The disease may present with a wide spectrum of clinical manifestations, including pneumonia, visceral abscesses, and septicemia. Neurological complications are uncommon but have been reported, with dural venous sinus thrombosis being a particularly rare manifestation.

We describe a case of a 55-year-old Malay woman with underlying diabetes mellitus and hypertension who presented with a one-week history of fever, confusion, back pain, and dyspnoea. Blood cultures grew *Burkholderia pseudomallei*, and imaging demonstrated disseminated infection with multiple splenic abscesses and pulmonary septic emboli, as well as dural venous sinus thrombosis.

The patient was initially treated with intravenous meropenem, followed by ceftazidime and oral trimethoprim-sulfamethoxazole as part of eradication therapy. Therapeutic anticoagulation with enoxaparin was also initiated. Follow-up imaging demonstrated significant resolution of the visceral abscesses and pulmonary lesions.

## Introduction

Melioidosis is a potentially life-threatening infectious disease caused by the Gram-negative bacillus *Burkholderia pseudomallei*. The disease is endemic in many tropical regions, particularly in Southeast Asia and northern Australia, and remains an important cause of community-acquired sepsis in countries such as Malaysia. Diabetes mellitus is the most important risk factor for melioidosis, although other conditions associated with impaired immunity may also increase susceptibility [1].

The clinical manifestations of melioidosis are highly variable and may range from localized infection to disseminated disease. Pneumonia is the most common presentation, while internal organ abscesses, particularly involving the spleen and liver, are also frequently reported [2]. In a small proportion of cases, the infection may involve the central nervous system, resulting in neurological melioidosis. Neurological manifestations include encephalomyelitis, cerebral abscess, cranial nerve palsies, and, rarely, septic cerebral venous thrombosis [3].

Dural venous sinus thrombosis (DVST) is an uncommon but serious complication that may lead to raised intracranial pressure, cerebral edema, and neurological dysfunction. Infection-related DVST is rare and may present diagnostic and therapeutic challenges. In this report, we describe a case of disseminated melioidosis involving the lungs and spleen complicated by DVST, highlighting the importance of early recognition and prompt management of this unusual manifestation.

## Case presentation

A 55-year-old Malay woman with underlying diabetes mellitus and hypertension presented to the hospital with a 1-week history of fever, back pain, dyspnoea, and reduced oral intake. On arrival, she was febrile with a temperature of 38 °C and tachypneic with a respiratory rate of 32 breaths per minute. Oxygen saturation was 98% while receiving oxygen via a Venturi mask at 40%. On chest auscultation, coarse crepitations were heard over the bilateral lower lung zones. Based on the presenting symptoms and examination findings, she was initially managed as a case of community-acquired pneumonia and was started on intravenous amoxicillin-clavulanate 1.2 g every 8 hours.

On day 3 of hospitalization, the patient developed acute confusion with a Glasgow Coma Scale score of E4V4M6. This was accompanied by a sudden episode of oxygen desaturation and a high-grade fever reaching 40 °C. Due to clinical deterioration, oxygen therapy was escalated to a high-flow mask, and antimicrobial treatment was escalated to intravenous meropenem. Blood cultures obtained on admission subsequently grew *Burkholderia pseudomallei*, identified using matrix-assisted laser desorption/ionization time-of-flight (MALDI-TOF), therefore confirming the diagnosis of melioidosis. The isolate was susceptible to meropenem, ceftazidime, and trimethoprim-sulfamethoxazole. An ultrasound examination of the abdomen demonstrated multiple splenic abscesses. A plain computed tomography (CT) scan of the brain was performed and showed no evidence of intracranial haemorrhage.

**Figure 1:**
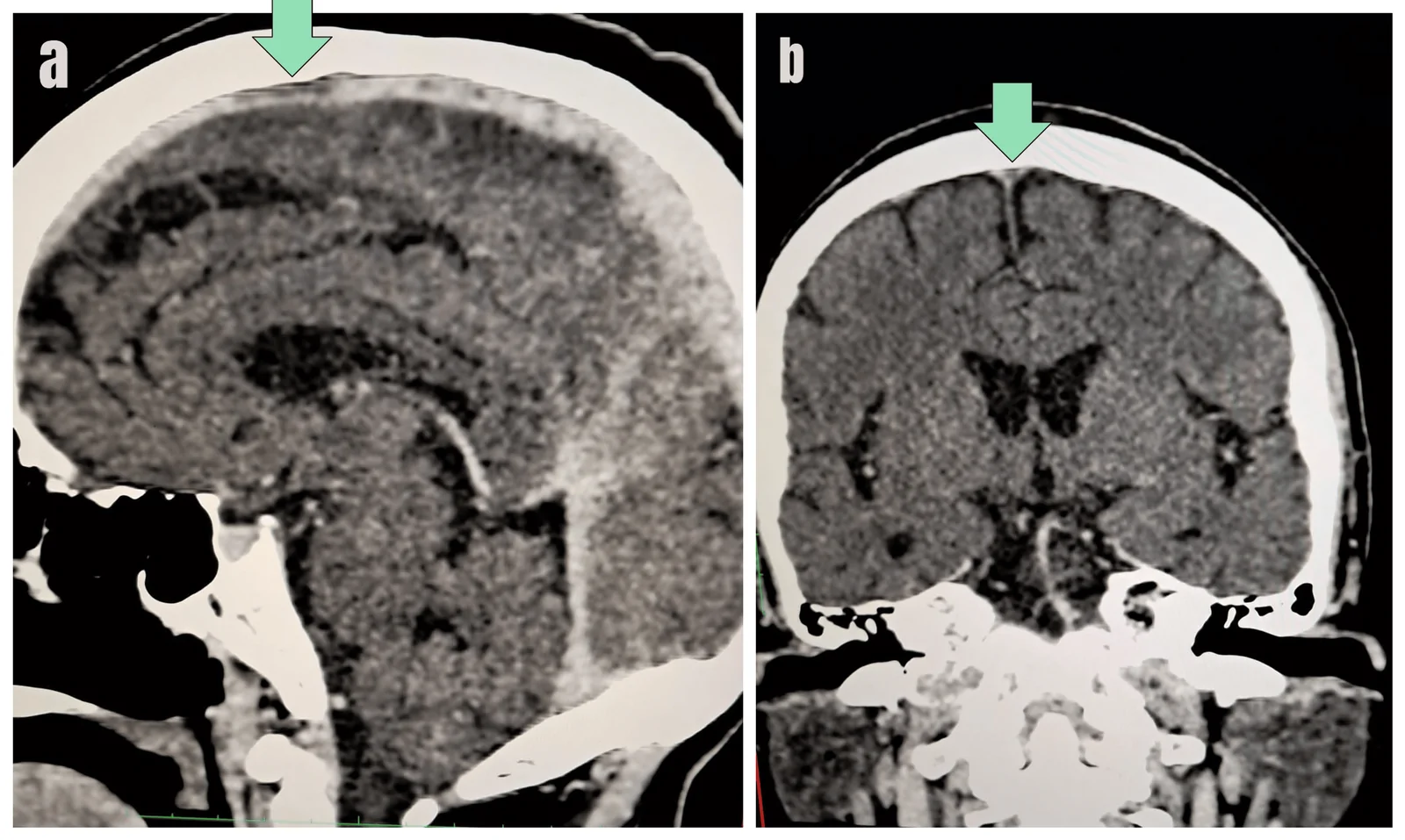
Contrast-enhanced CT scan of the brain (a) Sagittal and (b) coronal views reveal a well-defined intraluminal filling defect within the superior sagittal sinus (indicated by green arrows), representing a non-enhancing clot consistent with acute dural venous sinus thrombosis.

**Figure 2:**
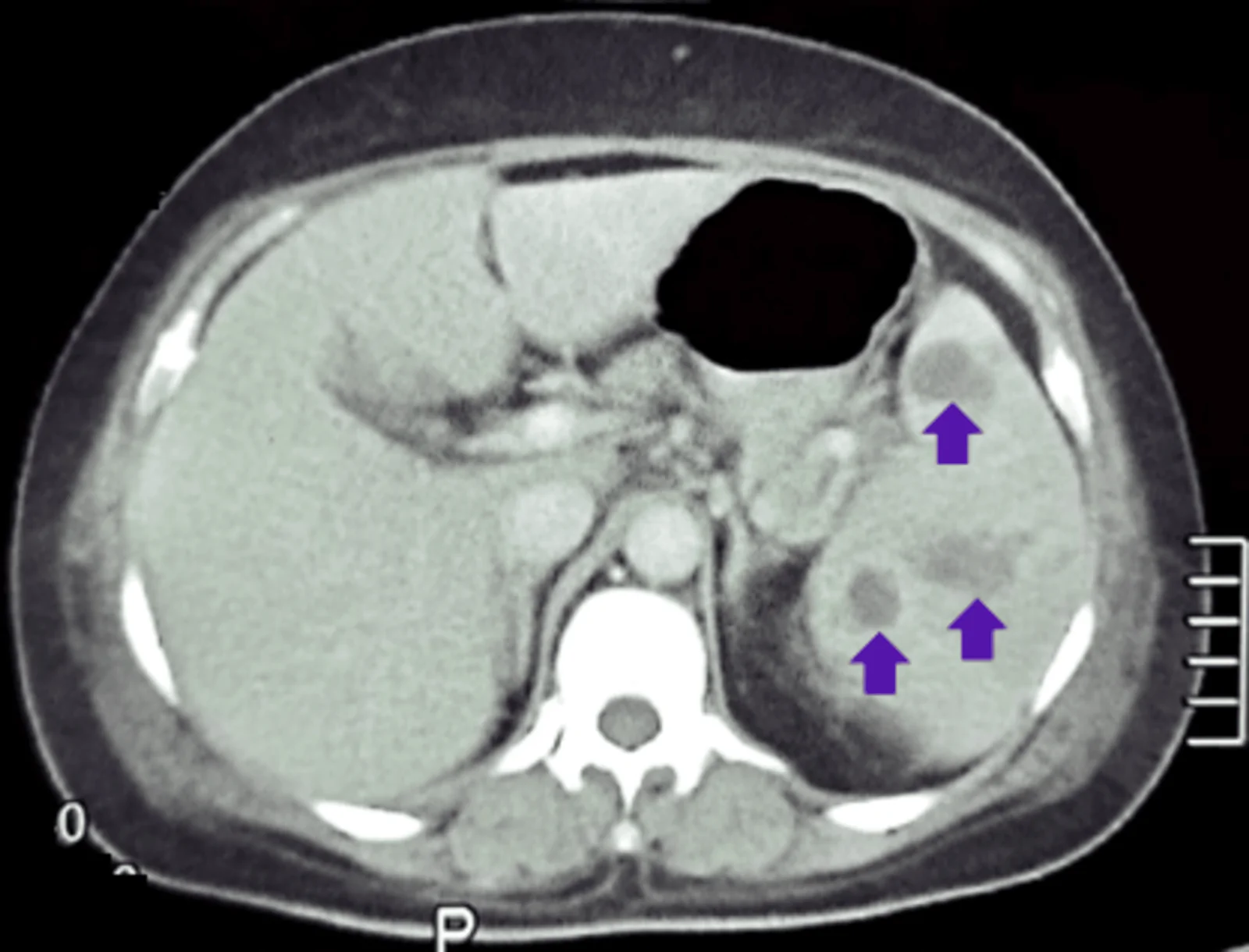
Abdominal CT scan showing multiple splenic abscesses

Given the new onset of altered mental status, further imaging was undertaken. On day 4 of admission, contrast-enhanced CT (Canon Aquilion ONE, Hospital Sultanah Aminah) of the brain, thorax, abdomen, and pelvis revealed a filling defect within the superior sagittal sinus, consistent with dural venous sinus thrombosis (Figure 1). The scan also demonstrated multiple bilateral pulmonary nodules with features suggestive of septic emboli, as well as several splenic lesions compatible with loculated abscesses (Figure 2). These findings supported the diagnosis of disseminated melioidosis involving the lungs and spleen, complicated by central nervous system involvement in the form of septic dural venous sinus thrombosis.

As her clinical condition began to improve and the microbiological diagnosis was established, antimicrobial therapy was de-escalated to intravenous ceftazidime to complete a total of four weeks of intensive therapy. Therapeutic anticoagulation with enoxaparin was initiated for the dural venous sinus thrombosis, and oral trimethoprim-sulfamethoxazole was also commenced.

The patient demonstrated progressive clinical improvement during hospitalization and gradually regained full orientation and functional mobility. She eventually became ambulatory with no focal neurological deficits. A follow-up contrast-enhanced CT scan of the thorax, abdomen, and pelvis performed after four weeks of treatment demonstrated a favorable radiological response, with a reduction in the size and number of bilateral pulmonary nodules, regression of the splenic abscesses, and complete resolution of the dural venous sinus thrombosis.

She was subsequently discharged home in stable condition with a plan for continued oral eradication therapy using trimethoprim-sulfamethoxazole and a three-month course of a direct oral anticoagulant for treatment of the dural venous sinus thrombosis. Close outpatient follow-up was arranged to monitor treatment response and ensure adherence to long-term antibiotic therapy.

## Discussion

Melioidosis is an endemic infection in many tropical regions, particularly in Southeast Asia and northern Australia. It is caused by *Burkholderia pseudomallei*, a Gram-negative environmental bacterium commonly found in soil and surface water. Infection occurs through inoculation, inhalation, or ingestion, and the clinical spectrum ranges from localized infection to fulminant septicaemia with multiorgan involvement. Melioidosis is well-recognized for its diverse clinical manifestations and its ability to mimic other conditions, earning it the name “the great mimicker” [1,2].

This case illustrates severe systemic dissemination of melioidosis complicated by a rare neurological manifestation. The patient’s pre-existing diabetes mellitus was a significant predisposing factor for disseminated infection, particularly the development of splenic abscesses and pulmonary septic emboli, both of which are characteristic manifestations of disseminated melioidosis [1]. The most notable and unusual finding in this case was the development of sagittal sinus thrombosis, a complication that has rarely been described in association with melioidosis.

Neurological involvement in melioidosis, also referred to as neuromelioidosis, is relatively uncommon, accounting for approximately 3-5% of cases. The most frequently reported manifestations include brain abscess, encephalomyelitis, and cranial nerve palsies [3]. In contrast, dural venous sinus thrombosis (DVST) is exceedingly rare and may easily be overlooked if appropriate neuroimaging is not performed [4]. The pathogenesis of DVST in melioidosis is likely multifactorial. *Burkholderia pseudomallei* may induce endothelial injury and inflammation, resulting in a prothrombotic state. Furthermore, the systemic inflammatory response associated with severe infection and sepsis may promote hypercoagulability and coagulopathy [5,6]. The filling defect within the superior sagittal sinus visualized on contrast-enhanced CT of the brain was crucial in establishing the diagnosis.

Dural venous sinus thrombosis is a rare but increasingly recognized neurological complication of melioidosis. The first documented case was reported by Niyasom et al. in 2006, describing superior sagittal sinus thrombosis in a patient with septicaemic melioidosis [4]. Bahuleyan et al. described a patient with cranial melioidosis complicated by cerebral venous sinus thrombosis in the setting of skull osteomyelitis and multiple intracranial abscesses [6]. More recently, Nayak et al. reported chronic pachymeningitis with dural venous sinus thrombosis as an unusual presentation of cranial melioidosis [7].

Management of such cases requires a multidisciplinary approach. Severe disseminated melioidosis with neurological involvement necessitates aggressive intravenous antimicrobial therapy, with carbapenems such as meropenem being the treatment of choice during the intensive phase [8]. In addition to antimicrobial therapy, therapeutic anticoagulation was initiated in this patient to prevent propagation of the venous thrombosis and further neurological deterioration [9]. The patient’s subsequent clinical improvement suggests that the combined approach of targeted antimicrobial therapy and anticoagulation was effective. Completion of the eradication phase with prolonged oral trimethoprim-sulfamethoxazole remains essential to reduce the risk of relapse.

## Conclusions

This case highlights several important clinical lessons. First, clinicians practicing in endemic regions should maintain a high index of suspicion for melioidosis in patients presenting with sepsis and multiple internal organ abscesses, particularly in those with diabetes mellitus. Second, neurological symptoms in disseminated melioidosis warrant prompt neuroimaging to exclude uncommon complications such as cerebral abscess or venous sinus thrombosis.
